# Aldehyde Dehydrogenase 1B1 Is Implicated in DNA Damage Response in Human Colorectal Adenocarcinoma

**DOI:** 10.3390/cells11132017

**Published:** 2022-06-24

**Authors:** Ilias Tsochantaridis, Alexandros Kontopoulos, Georgia-Persephoni Voulgaridou, Margaritis Tsifintaris, Charisios Triantafyllou, Aglaia Pappa

**Affiliations:** Department of Molecular Biology & Genetics, Faculty of Health Sciences, Democritus University of Thrace, 68100 Alexandroupolis, Greece; iliatsoc@gmail.com (I.T.); alexkntp98@gmail.com (A.K.); gvoulgar@mbg.duth.gr (G.-P.V.); mtsifintaris@gmail.com (M.T.); charisiostriantafyllou@gmail.com (C.T.)

**Keywords:** aldehyde dehydrogenases (ALDH), aldehyde dehydrogenase (ALDH1B1), DNA damage response (DDR), DNA repair, p53, phospo-p53, γH2AX, HT29, colorectal adenocarcinoma

## Abstract

Aldehyde dehydrogenase 1B1 (ALDH1B1) has been correlated with colorectal tumorigenesis and is considered a potential biomarker for colon cancer. Its expression has been associated with attenuation of the cell cycle in the G2/M phase and resistance to DNA damaging agents. The present study examines the role of ALDH1B1 in DNA damage response (DDR) in human colorectal adenocarcinoma. To this end, we utilized an isogenic HT29 cell line pair differing in the expression of ALDH1B1. The overexpression of ALDH1B1 was related to the translational upregulation of the total and phosphorylated (at ser15) p53. Comet and apoptosis assays revealed that the expression of ALDH1B1 protected HT29 cells from etoposide-induced DNA damage as well as apoptosis, and its overexpression led to increased constitutive phosphorylation of H2AX (at ser139). Furthermore, the expression profile of a variety of DNA damage signaling (DDS)-related genes was investigated by utilizing the RT^2^ profiler™ PCR array. Our results demonstrated that ALDH1B1 triggered a transcriptional activation of several DNA repair-related genes (*MRE11A*, *PMS1*, *RAD18* and *UNG*). Finally, Spearman’s rank correlation coefficient analysis in 531 publicly available colorectal adenocarcinoma clinical samples indicated the statistically significant positive correlation between ALDH1B1 and DDR and repair genes or proteins, such as APEX1, FEN1, MPG, UNG, XRCC1, DDB1, XPC, CIB1, MRE11, PRKDC, RAD50, RAD21, TP53BP1, XRCC6 and H2AX. Collectively, our results suggest that ALDH1B1 may play an essential role in the DDR and DNA repair processes. Further studies on ALDH1B1 will elucidate its precise role in DDR.

## 1. Introduction

Aldehyde dehydrogenases (ALDHs) are NAD(P)^+^-dependent enzymes that catalyze the oxidation of endogenous and exogenous aldehydes to their corresponding carboxylic acids [1]. ALDHs are important enzymes for metabolic, cyto-protective, antioxidant and homeostatic functions; they are categorized into 11 families and 4 sub-families and exhibit different chromosomal locations, expression patterns, cellular localization, substrate specificity and tissue distribution [2,3].

Aldehyde dehydrogenase 1B1 (ALDH1B1), a member of the ALDH superfamily, is a mitochondrial enzyme (517 amino acids) that detoxifies lipid peroxidation by-products malondialdehyde (MDA) and 4-hydroxy-nonenal (4-HNE) and metabolizes nitroglycerin and all-trans retinaldehyde [2]. ALDH1B1 exhibits 72% and >60% sequence homology with ALDH2 and ALDH1A1, respectively [4,5]. Recent studies suggest that ALDH1B1 is associated with pancreatic stem cells [6] and beta cell development [7] and is involved in various cellular signaling pathways such as PI3K/Akt, Wnt/β-catenin and Notch, demonstrating its potential correlation with the CSC phenotype [8]. Moreover, studies have reported that ALDH1B1 could be a novel biomarker for detecting colorectal cancer [9] and may be a modulator of pancreatic cancer [10,11].

Colorectal cancer (CRC) is estimated to be the third most common type of cancer and cause of cancer-related deaths in the USA [12]. Recent studies have indicated the ALDH1B1 association with colorectal tumorigenesis [2,8,9,13,14,15]. More specifically, Want et al. showed that ALDH1B1 is closely related to CRC, as its expression was substantially increased in colorectal adenomas and adenocarcinomas compared to normal and cancer-adjacent tissues [15]. Furthermore, we have previously demonstrated that ALDH1B1 expression triggered: morphological alterations associated with decreased proliferative potential and clonogenicity; cell cycle attenuation at the G2/M phase; enhanced chemo-resistance against doxorubicin, 5-fluorouracil (5-FU) and etoposide; ZEB1-related EMT induction; increased migratory potential; and a p53- and p21-regulated G2/M cell cycle arrest in HT29 cells [2].

Given the ALDH1B1-driven cell cycle attenuation at the G2/M phase, and chemoresistance against DNA-damage agents previously observed in human colorectal adenocarcinoma cells [2], we hypothesize that ALDH1B1 plays an important role in DDR mechanisms and the development of colon cancer. In the present study, we tested this hypothesis by utilizing an isogenic cell line pair of human colorectal adenocarcinoma cells (HT29), differing only in the ALDH1B1 expression previously established by lentiviral stable transfection [2], and publicly available data on colon or rectal adenocarcinoma clinical samples [16,17]. We demonstrated that ALDH1B1 is associated with decreased etoposide-induced DNA damage and apoptosis and with increased constitutive phosphorylation of H2AX. Furthermore, the expression of ALDH1B1 triggered altered profiles of various DDS-related genes, which was further validated by analyzing 531 publicly available colon and rectal adenocarcinoma clinical samples. Our data support that an important role of ALDH1B1 is conferring cell survival advantages under conditions of DNA damage.

## 2. Materials and Methods

### 2.1. Materials

A human colorectal cancer HT29 cell line was obtained from ATCC (Manassas, VA, USA). Culture medium, fetal bovine serum (FBS), penicillin/streptomycin (100X) solution and trypsin were from Biosera (Boussens, France). Cell culture flasks and plates, Falcon and Eppendorf tubes were from SPL Life Sciences (Geumgang-ro 2047 beon-gil, Naechon-Myeon, Pocheon-si, Gyeonggi-do 487 835, Korea). NucleoZOL was from Macherey-Nagel (Düren, Germany), primers, dNTPs, random hexamers and PrimeScript reverse transcriptase were purchased from Invitrogen (Thermo Fischer Scientific, Waltham, MA, USA), and the KAPA SYBR Fast Master Mix was from Kapa Biosystems (Hoffmann-La Roche, Basel, Switzerland). Propidium iodide (PI) was purchased from Biotium (Landing Parkway Fremont, CA, USA). APC Annexin V was from Biolegend (San Diego, CA, USA). For protein estimation, the Pierce™ BCA Protein assay was purchased from Thermo Scientific (Rockford, IL, USA), whereas the polyvinylidene difluoride (PVDF) membranes and chemiluminescence reagents were obtained from Millipore (Bedford, MA, USA). The 40% acrylamide was from Bio-rad (Hercules, CA, USA). Primary antibodies anti-p53 (1C12), anti-phospho-p53 (ser15), anti-phospho H2AX (ser139), anti-GAPDH (14C10) and anti-histone H3 (D1H2) were obtained from Cell Signaling Technology (Boston, MA, USA). Goat anti-rabbit and mouse IgG horseradish peroxidase conjugated antibodies were from Millipore (Burlington, MA, USA). The RT^2^ Profiler PCR array for the DNA damage signaling pathway was purchased from Qiagen (Venlo, The Netherlands).

### 2.2. Cell Culture

The human colorectal cancer cell line (HT29) was maintained as described previously [2].

### 2.3. Protein Extraction, Cell Lysates Preparation and Western Immunoblotting

Protein extraction, cell lysate preparation and western immunoblotting were performed as described previously with minor modifications [2]. In brief, cells were lysed with RIPA (50 mM NaCl, 50 mM Tris pH 8.0, 1% Nonidet P40 (NP-40), 0.25% sodium deoxycholate, 0.25% SDS, 1 mM EDTA) or fractionation lysis buffer (10 mM HEPES, 10 mM KCl, 0.1 mM EDTA, 1.5 mM MgCl_2_, 0.2% NP-40) and supplemented with protease inhibitors (100 μg/mL PMSF, 0.5 μg/mL leupeptin, 0.5 μg/mL aprotinin, 1 μg/mL pepstatin A) and a phosphatase inhibitor cocktail. Whole cell lysates were sonicated (5 s, twice) and incubated for 30 min at 4 °C, pipetting up and down or vortexing every 10 min. Samples were centrifuged at 12,500 rpm for 15 min (4 °C) or at 1000× *g* for 10 min (4 °C) for the preparation of cytosolic (supernatant) and nuclear (pellet) fractions. Whole cell lysates (40 μg) or nuclear (15 μg) were subjected to SDS-PAGE and western immunoblotting and incubated with primary and secondary antibodies as described previously [2]. Band intensity was quantified by scanning densitometry using the Image J software (1.44n, National Institute of Health, Bethesda, MD, USA).

### 2.4. Flow Cytometry Analysis

#### 2.4.1. Flow Cytometric Analysis of Total p53 Expression

Total p53 expression was assessed through flow cytometry as described previously for p21 expression [2]. Histograms of p53 median fluorescence intensity were generated by Flowjo software (v.10 FlowJo LLC, Ashland, OR, USA).

#### 2.4.2. Annexin V—Propidium Iodide (PI) Double Staining

Apoptotic cells were detected by using Annexin V conjugated with allophycocyanin (APC) and PI double staining according to the manufacturer’s instructions. Briefly, HT29 cells were plated in 90 × 20 mm-well plates (1.5 × 10^5^ cells/plate) and treated with etoposide (50 μΜ and 75 μΜ) for 24 h. Then, cells were collected (dead cells were also kept), washed twice with ice-cold PBS, counted and resuspended to a final concentration of 10^6^ cells/mL in 1X binding buffer (10× binding buffer: 0.1 M Hepes/NaOH (pH 7.4), 1.4 M NaCl and 25 mM CaCl_2_). Next, 100 μL from each sample were transferred to a 5 mL FACS tube, in which 5 μL Annexin V-APC were added. Samples were incubated in the dark for 13 min, and then 3 μL propidium iodide (PI) were added together with 400 μL 1× binding buffer. Samples were analyzed in an Attune NxT flow cytometer (Thermo Fisher Scientific) and were processed using Flowjo v.10 (FlowJo LLC) software.

### 2.5. Single Cell Gel Electrophoresis Assay (Comet Assay)

HT29 8 × 10^3^ cells (2 × 10^4^ cells/mL of PBS w/o Ca^2+^/Mg^2+^) were suspended in 1.2 mL low-melting point agarose and placed onto super frosted glass microscope slides pre-coated with 1% agarose. Then, the comet assay was conducted as described previously [18,19,20]. The overall DNA damage was estimated in arbitrary units as previously described [19].

### 2.6. RT^2^ Profiler^TM^ PCR Array for DNA Damage Signaling Pathway

Total RNA (0.5 μg) was extracted by utilizing the NucleoZOL reagent, and cDNA synthesis was performed with a RT^2^ first strand kit according to the manufacturer’s instructions. A volume of 1248 μL RNase-free water and 1350 μL 2× RT^2^ SYBR green master mix was then added to the 102μL reaction mixture. Twenty-five (25) μL of this mixture were used per well in the 96-well PCR array (RT^2^ Profiler PCR Array for Human DNA Damage Signaling Pathway/PAHS-029ZA). The information of the genes included in the PCR array is included in Table S1.

Reactions were performed in an Applied Biosystems^®^ 7500 model under the program, which is described previously [20], and the analysis was conducted through https://geneglobe.qiagen.com/us/analyze (accessed on 27 July 2021).

### 2.7. Data Acquisition and Bioinformatics Analysis

Data from colorectal adenocarcinoma patient samples (531 patients with colon and/or rectal adenocarcinoma) were obtained from The Cancer Genome Atlas (TCGA) database (downloaded from cBioportal.org, accessed on 1 February 2022) [16,17] and used to determine the correlation between ALDH1B1 and 84 DDS-related genes and proteins. TCGA mainly contains data on cancer tissues, including data on 33 types of tumors. These data are comprehensive, covering RNA-seq, mass spectroscopy, miRNA mutation, methylation and other data. The analysis was performed for all available RNA-seq and mass spectrometry data. For the purpose of the study, we utilized 531 colon and rectal cancer clinical samples with available information on mRNA expression levels and 77 cancer clinical samples with available information on protein expression levels. Spearman’s correlation analysis was performed for all available mass spectrometry (77 samples) and log_2_x + 1 transformed RNA-seq (531 samples) data. The correlation heat maps were conducted using R language and the corrplot package.

### 2.8. Statistical Analysis

Statistical analysis was performed as described previously [20]. Spearman’s rank correlation coefficient was used to ascertain the strength and direction of a relationship between *ALDH1B1* gene expression and DDS-related genes or the ALDH1B1 protein and DDS-related proteins. A *p* value < 0.05 was considered as statistically significant.

## 3. Results

### 3.1. ALDH1B1 Expression Leads to Increased Constitutive Phosphorylation of p53 (at ser15) in HT29 Cells

**Figure 1:**
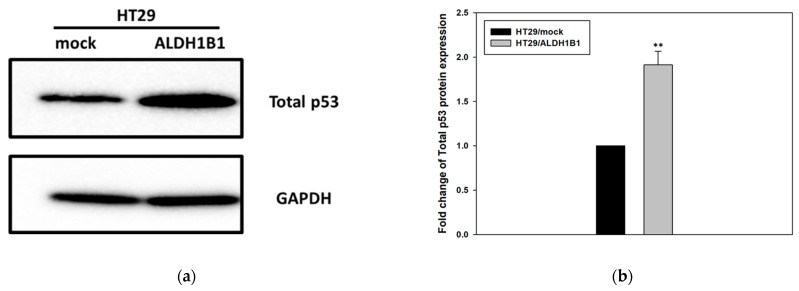

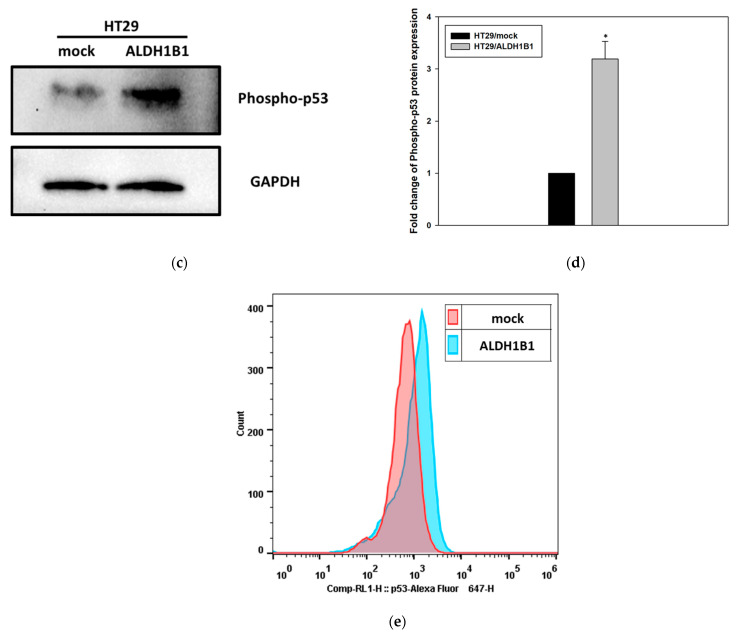
**ALDH1B1 induces the upregulation of the total and phosphorylated form of p53 protein in HT29 cells.** (**a**,**c**) Forty (40) μg of HT29/ALDH1B1 and HT29/mock cell extracts were subjected to western blot analysis against total p53 and p53 phosphorylation. GAPDH was used as a control for equal loading. (**b**,**d**) The translational expression levels of total and phosphorylated (ser15) p53 were ~2-fold and >3-fold, respectively; they were higher in ALDH1B1-expressing cells compared to mock control HT29 cells. (**e**) The ALDH1B1 histogram (light-blue color) had appreciably right-shifted in comparison with HT29/mock cells (pink color), demonstrating the increased total p53 expression levels in ALDH1B1-overexpressing cells. At least 20,000 events were analyzed through flow cytometry, and the median fluorescence intensity for total p53 was determined in both cell lines (Table 1). The above graphs are representative of an experiment. At least three independent experiments were performed for each condition. * *p* < 0.05, ** *p* < 0.01.

In a previous study we demonstrated that ALDH1B1 is associated with a G2/M cell cycle arrest in HT29 cells overexpressing ALDH1B1 [2]. This observation prompted us to evaluate the translational expression levels of the phosphorylated, at serine 15 residue, form of p53 protein. As indicated in Figure 1 and Table 1, western immunoblotting and/or flow cytometry revealed that the expression of total p53 and phopsho-p53 protein was found to be up-regulated (~2-fold for total and >3-fold for phospho-p53) in the ALDH1B1-overexpressing cells compared to HT29/mock cells.

### 3.2. ALDH1B1 Protects HT29 Cells from Etoposide-Induced DNA Damage

**Figure 2:**
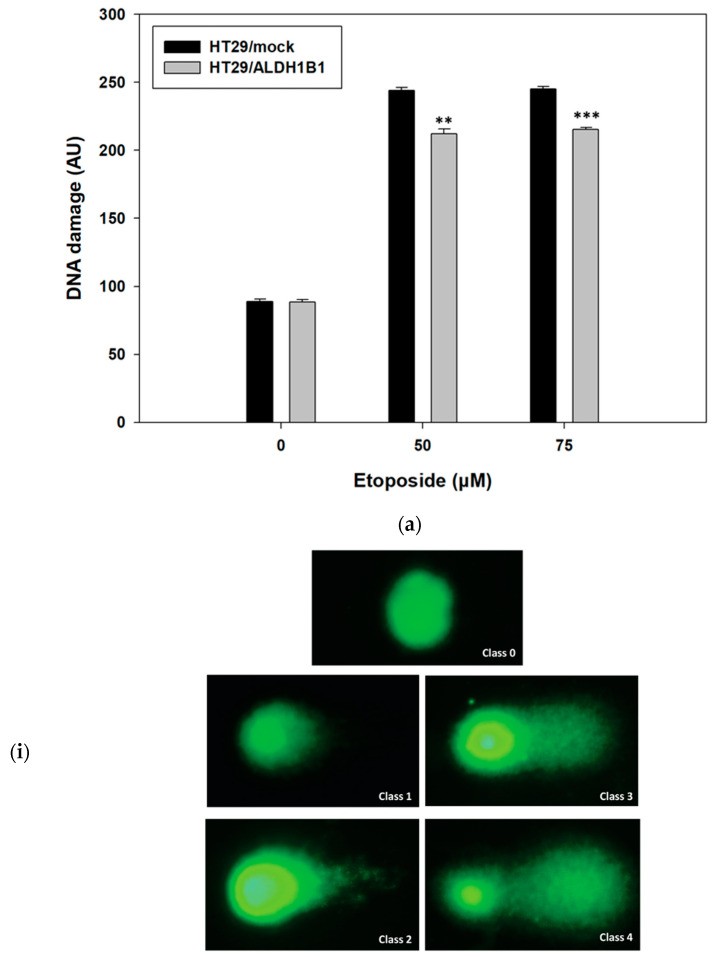

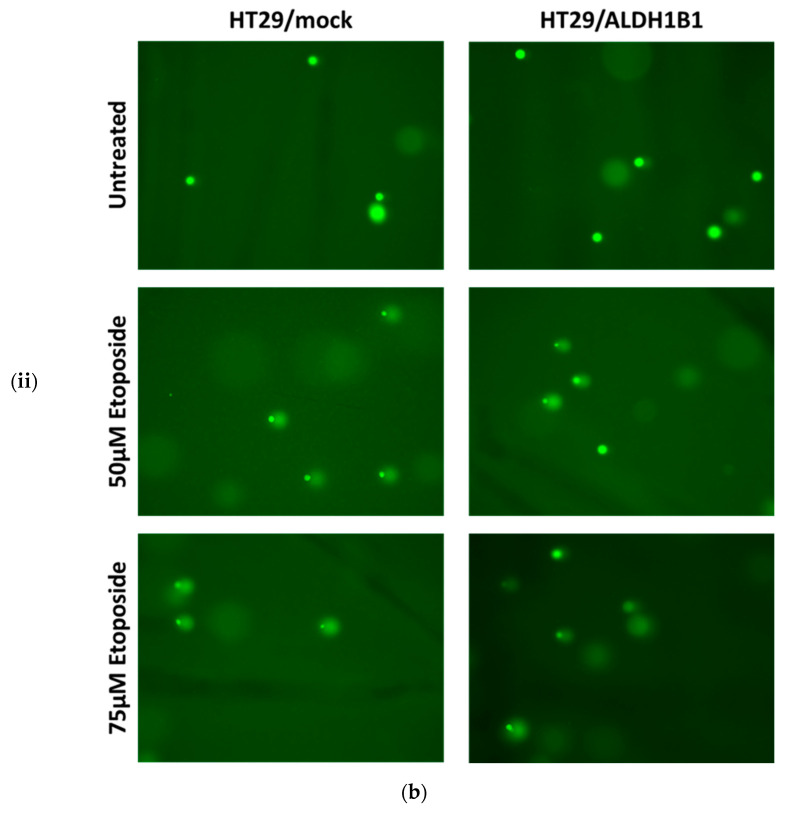
**Detection of DNA damage in etoposide-treated HT29/ALDH1B1 and HT29/mock cells by a comet assay.** (**a**) ALDH1B1 maintains etoposide-induced DNA damage in HT29/ALDH1B1 cells at significantly lower levels compared to the HT29/mock cells. The scored AU (arbitrary units), represent the extent of DNA damage in HT29/mock and HT29/ALDH1B1 cells following 24 h of incubation with the indicated concentrations of etoposide. (**b-i**). Representative comets of various classes 0, 1, 2, 3 and 4 are shown. For the classification of DNA damage in individual cells, the size and integrity of the comet’s head, the intensity of the tail and the head/tail analogy were taken into account. (**b-ii**) Representative comet images from HT29/mock and HT29/ALDH1B1 cells under treatment with 0, 50 and 75 μM etoposide are shown. Results are shown as mean ± S.D. At least three independent experiments were performed for each condition. ** *p* < 0.01, *** *p* < 0.001.

Previously, we demonstrated that ALDH1B1 confers resistance against etoposide [2], an agent that causes DNA damage. In the current study, we compared the etoposide-induced DNA damage levels between HT29/mock and ALDH1B1-overexpressing cells. HT29/ALDH1B1 and HT29/mock cells were incubated for 24 h with etoposide (50 μΜ and 75 μM) (Figure 2). DNA damage was evaluated under alkaline conditions by using a comet assay. Interestingly, ALDH1B1-overexpressing cells exhibited significantly lower DNA damage in comparison with HT29/mock control cells at both tested concentrations.

### 3.3. ALDH1B1 Protects HT29 Cells from Etoposide-Induced Apoptosis

**Figure 3:**
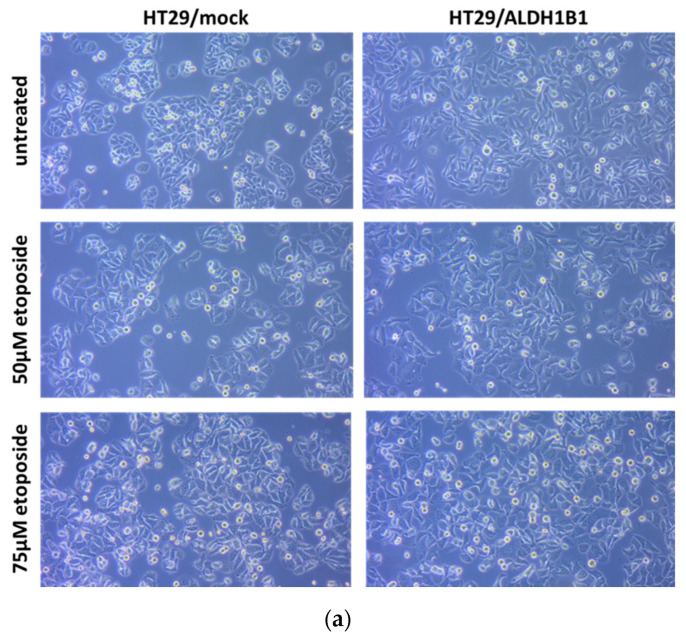

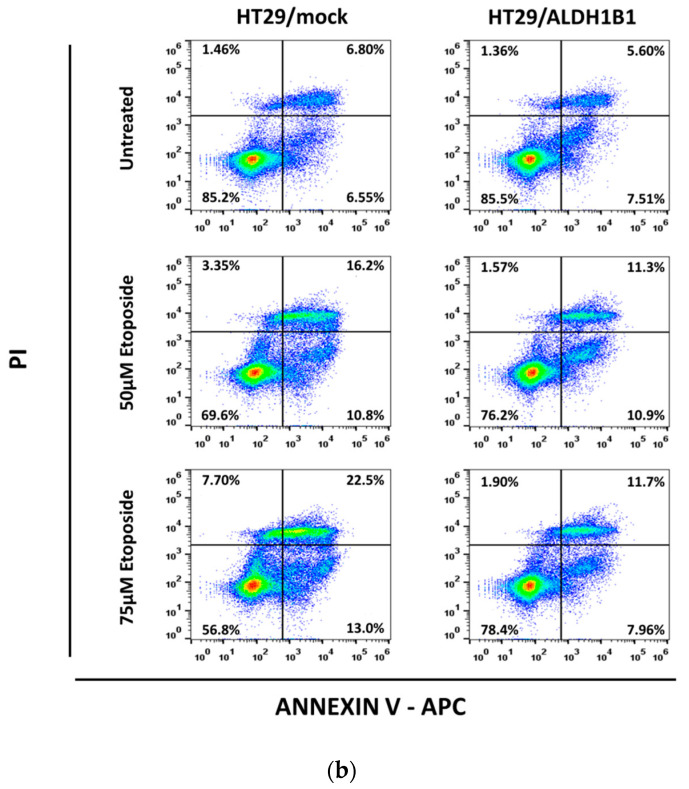
Flow cytometry analysis using an Annexin V/PI double-staining assay in a HT29 isogenic cell line pair under normal conditions and following etoposide treatment. (**a**) Etoposide triggered significant morphological alteration in the isogenic cell line pair. (**b**) Cells were stained with Annexin V-APC and PI. Live cells are found in the lower left quadrant (Annexin V and PI negative), early apoptotic cells are indicated in the lower right quadrant (Annexin V positive and PI negative), late apoptotic cells are demonstrated in the upper right quadrant (Annexin V and PI positive) and dead cells are shown in upper left quadrant (Annexin V negative and PI positive). The colors represent the collection of events/cells with the same intensity detected during flow cytometry. Blue represents low intensity (single events/cells) whereas red represents high intensity.

The increased phosphorylation of p53 in the ALDH1B1-overexpressing cells prompted us to further assess the apoptotic process in the HT29 isogenic cell line pair. P53 is important for DNA damage-induced apoptosis and its phosphorylation at ser15 residue is significant for cell cycle arrest [21]. DNA damage response is a cellular process, which involves cell cycle arrest, DNA repair and ineffective repair-related cell death [22]. For this purpose, we performed flow cytometry analysis detecting PI and Annexin V-APC in HT29 cells under normal and etoposide conditions. PI binds to the DNA, whereas Annexin V binds to PS, which is normally located in the inner side of the cell membrane. During apoptosis, PS is translocated to the outer surface of the membrane, and Annexin V binds to PS, acting as a marker of apoptosis. HT29/ALDH1B1 and HT29/mock cells are treated with etoposide for 24 h before assessing apoptosis. As shown in Figure 3, etoposide triggered significant morphological alterations and induced apoptosis in HT29 cells. The percentages of cells during each stage of apoptosis under normal and etoposide conditions are demonstrated in Table 2. The percentage (%) of live cells (unstained, Annexin V^-^–PI^-^) was higher in ALDH1B1-expressing cells than mock control cells under normal and etoposide conditions. Furthermore, early and late apoptotic cells were significantly lower in HT29/ALDH1B1 cells compared to mock control cells at both etoposide concentrations tested (50 μΜ and 75 μM).

### 3.4. ALDH1B1 Overexpression Leads to Increased Phosphorylation of H2AX (ser139) in HT29 Cells

**Figure 4:**
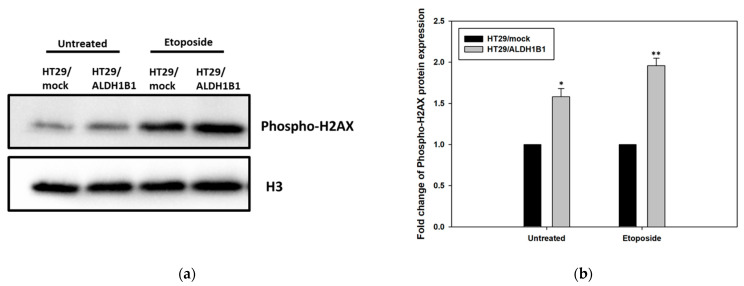
**Evaluation of H2AX phosphorylation (serine 139) (phospho-H2AX) in HT29/ALDH1B1 and HT29/mock cells.** (**a**) Forty (40) μg of HT29/ALDH1B1 and HT29/mock cell extracts were subjected to western blot analysis against H2AX phosphorylation. Histone H3 was used as a control for equal loading. (**b**) The protein expression levels of the phosphorylated (ser139) H2AX were >1.5-fold and >1.8-fold higher under normal and etoposide conditions, respectively, compared to HT29/mock cells. * *p* < 0.05, ** *p* < 0.01.

Next, we studied whether ALDH1B1 is capable of modulating the DNA damage response. To this end, we assessed H2AX phosphorylation under normal and treatment with etoposide conditions in HT29/ALDH1B1 and HT29/mock cells by employing western immunoblotting (Figure 4). Interestingly, the expression levels of phospho-H2AX were increased in the untreated and etoposide-treated (1 h) HT29/ALDH1B1 cells compared to HT29/mock cells. Our results demonstrated enhanced activation of H2AX in ALDH1B1-expressing cells in comparison with the mock control. Activation of H2AX is related to initiation of DNA repair as well as recruitment and accumulation of DNA damage response proteins [23].

### 3.5. HT29/ALDH1B1 Cells Exhibit the Differential Gene Expression Profile of DDR-Related Proteins Compared to HT29/Mock Cells

**Figure 5:**
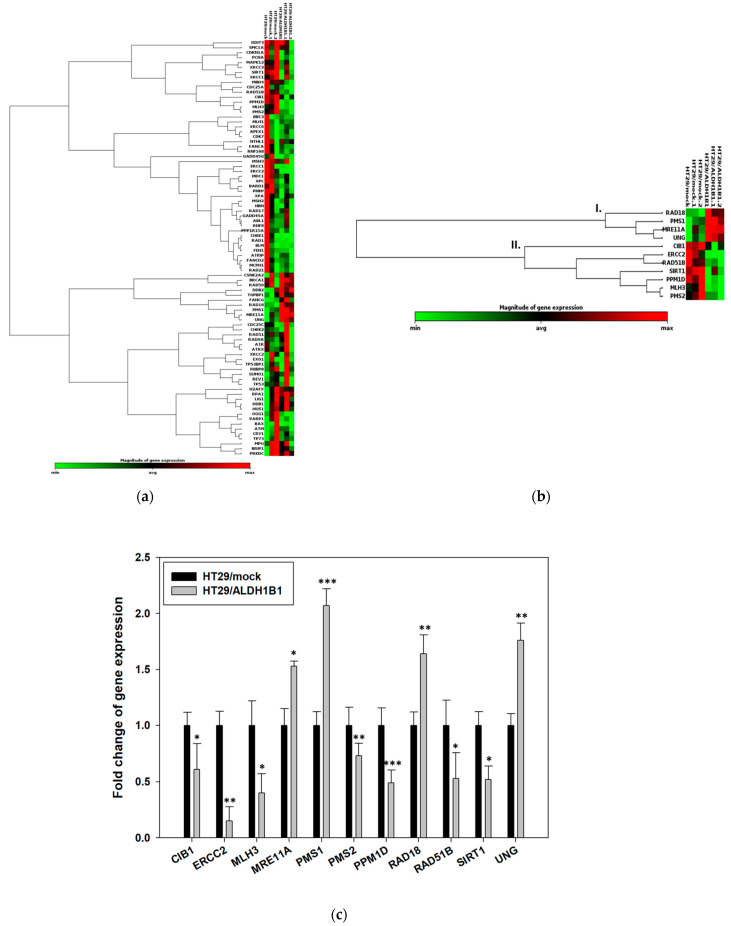
**ALDH1B1 promotes alterations in the gene expression profile of a panel of DNA damage signaling genes in HT29 cells.** Total RNA was extracted from the HT29 isogenic cell line pair, and the RT^2^ Profiler™ PCR Array for Human DNA Damage Signaling Pathway (PAHS-029ZA) was utilized to determine the expression profile of 84 different DNA damage signaling (DDS)-related genes. (**a**) The clustergram displays a heat map indicating the magnitude of gene expression along with a dendrogram, which organizes genes in proportion to their expression pattern. The color saturation indicates the magnitude of gene expression. Red squares indicate higher expression, black squares indicate no change, and green squares indicate lower expression. The x-axis shows the HT29/ALDH1B1 and HT29/mock samples of the three independent experiments, whereas the y-axis represents the PCR array genes. (**b**) Genes of cluster I (*MRE11A*, *PMS1*, *RAD18* and *UNG*) exhibited upregulation, whereas genes of cluster II (*CIB1*, *ERCC2*, *MLH3*, *PMS2*, *PPM1D*, *RAD51B* and *SIRT1*). (**c**) Genes *MRE11A, PMS1, RAD18* and *UNG* were found to be up-regulated in HT29/ALDH1B1 cells (fold change of expression (≥1.5)), whereas *CIB1*, *ERCC2*, *MLH3*, *PMS2*, *PPM1D*, *RAD51B* and *SIRT1* exhibited negative regulation in the ALDH1B1-overexpressing HT29 cells. Five different housekeeping genes were used for normalization (*ACTB*, *B2M*, *GAPDH*, *HPRT1* and *RPLP10*) of gene expression. Results are shown as mean ± S.D. Three independent experiments were performed for each sample. * *p* < 0.05, ** *p* < 0.01, *** *p* < 0.001.

HT29/ALDH1B1 cells demonstrated remarkable endurance against the etoposide-induced-DNA damage and cytotoxicity. Furthermore, phospho-H2AX was found to be up-regulated in the ALDH1B1-overexpressing cells under normal conditions and after treatment with etoposide. Next, we sought to analyze the expression of DDR-related genes in the isogenic HT29 cell line pair by performing the RT^2^ profiler Real-time PCR array. The expression levels of 84 different genes were quantified and compared between HT29/ALDH1B1 and HT29/mock cells by ΔΔCt method. PCR array results indicated that ALDH1B1-overexpressing HT29 cells exhibited significant differentiation in the transcriptional expression pattern of the genes tested compared to HT29/mock control cells (Figure 5). For further analysis, a threshold of fold regulation ≥1.5 or ≥0.5 and *p* value < 0.05 (for the samples with Ct < 30) was set, and a panel of 11 genes was further selected (Figure 5b,c). The clustergram of the selected genes (Figure 5b) shows the existence of two clusters (clusters I & II) with reverse expression patterns in the HT29 isogenic cell lines: cluster **I.** representing the up-regulated genes and cluster **II.** representing the down-regulated genes in HT29/ALDH1B1 cells compared to HT29/mock cells (Figure 5b). Specifically, *MRE11A, PMS1, RAD18 and UNG* (cluster I) were found to be substantially up-regulated (fold change ≥ 1.5), whereas downregulation was observed for *CIB1*, *ERCC2*, *MLH3*, *PMS2*, *PPM1D*, *RAD51B and SIRT1* (cluster II) (Figure 5b,c).

### 3.6. ALDH1B1 Is Associated with DDS-Related Genes and Proteins in 531 and 77 Colon and Rectal Adenocarcinoma Patient Samples, Respectively

To further validate our findings, we studied the relationship between ALDH1B1 expression (gene or protein levels) and each of the 84 different DNA damage response-related molecules (included in the RT^2^ profiler Real-time PCR array in our previous step) using publicly available data downloaded from the TCGA database and by employing Spearman’s rank correlation coefficient (Spearman’s rho) analysis. We utilized 531 cancer clinical samples with available RNA-Seq expression profile data and 77 cancer clinical samples with available mass spectroscopy profile data.

**Figure 6:**
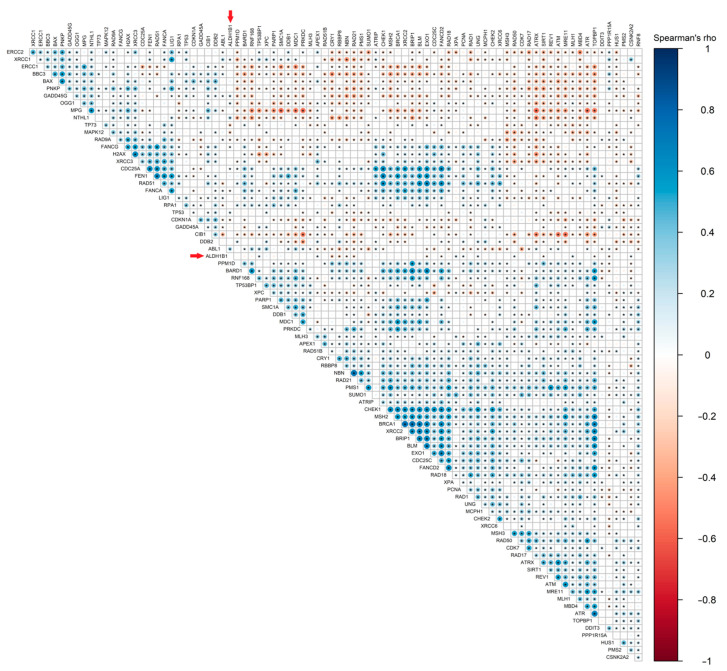
**Heat map showing the spearman correlation coefficient of pairwise comparison between DDS-related genes and *ALDH1B1* gene expression**. Correlation heatmap of gene expression data for 84 DDS-related genes (RNA-seq, TCGA). The heat map above demonstrates the magnitude of association between two genes of interest. The blue color represents a positive correlation, red indicates negative correlation and white means there is no correlation. The darker the color, the stronger the correlation. Red arrows demonstrate the ALDH1B1 gene in the correlation heat map. Statistically significant correlations (*p* < 0.05) are flagged with one star (*).

In 531 patients with colon or rectal adenocarcinoma, RNA-seq results indicated that *ALDH1B1* exhibits a substantial correlation with DNA repair genes. More specifically, *ALDH1B1* is statistically significantly positively correlated with *ABL1* (rho = 0.284, *p* < 0.0001), *APEX1* (rho = 0.124, *p* < 0.01), *CRY1* (rho = −0.218, *p* < 0.0001), *GADD45G* (rho = 0.170, *p* < 0.0001), *MAPK12* (rho = −0.208, *p* < 0.0001), *NTHL1* (rho = 0.237, *p* < 0.0001), *OGG1* (rho = 0.155, *p* < 0.001), *RAD21* (rho = 0.179, *p* < 0.0001), *RBBP8* (rho = −0.208, <0.0001), *TP53* (rho = 0.143, *p* < 0.001), *TP53BP1* (rho = 0.129, *p* < 0.01), *TP73* (rho = −0.204, *p* < 0.0001), *UNG* (rho = 0.121, *p* < 0.01), *XPA* (rho = 0.152, *p* < 0.001) and *XRCC1* (rho = 0.113, *p* < 0.01) (Figure 6, Supplementary Materials, Table S2).

**Figure 7:**
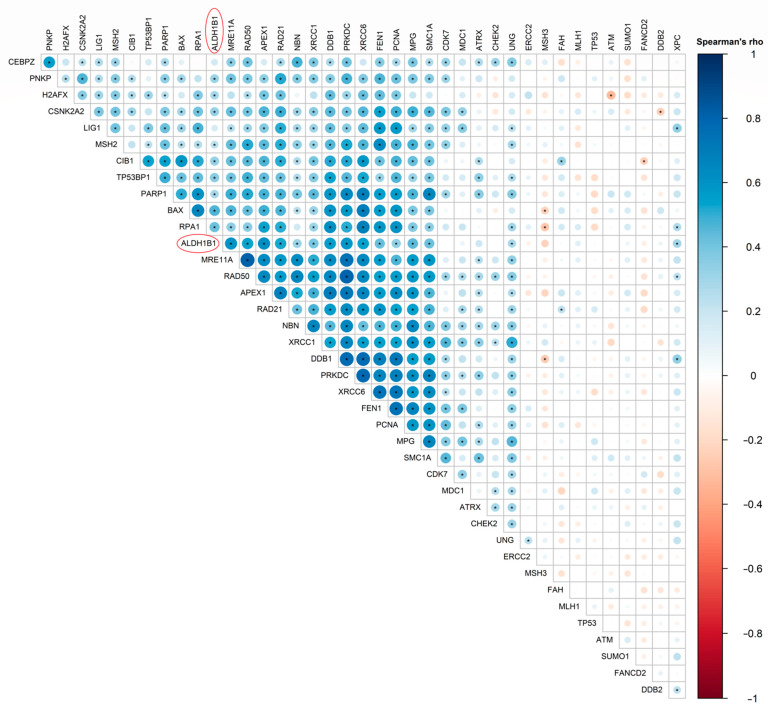
**Heat map showing the spearman correlation coefficient of pairwise comparison between DDS-related proteins and ALDH1B1 protein expression**. Spearman’s correlation heat map of the translational expression levels of 39 different DDS-related proteins compared to ALDH1B1 (MS data, TCGA). The heat map above represents the magnitude of association between two proteins of interest. The blue color represents a positive correlation, red indicates negative correlation and white means there is no correlation. The darker the color, the stronger the correlation. Red circles indicate ALDH1B1 protein in the correlation heat map. Statistically significant correlations (*p* < 0.05) are flagged with one star (*).

Moreover, we demonstrated that ALDH1B1 is strongly correlated with many proteins involved in single-strand damage and the double-strand breaks repair process. In particular, mass spec results in 77 colon and rectal adenocarcinoma patient samples revealed that ALDH1B1 exhibited a statistically significant positive correlation with base excision repair proteins [APEX1 (rho = 0.615, *p* < 0.0001), FEN1 (rho = 0.420, *p* < 0.001), MPG (rho = 0.445, *p* < 0.0001), UNG (rho = 0.275, *p* < 0.05) and XRCC1 (rho = 0.395, *p* < 0.001)], nucleotide excision repair proteins [DDB1 (rho = 0.556, *p* < 0.0001) and XPC (rho = 0.296, *p* < 0.01)], non-homologous end joining proteins (NHEJ) [CIB1 (rho = 0.330, *p* < 0.01), MRE11 (rho = 0.604, *p* value < 0.0001), PRKDC (rho = 0.584, *p* < 0.0001), RAD50 (rho = 0.532, *p* value < 0.0001), TP53BP1 (rho = 0.351, *p* < 0.01) and XRCC6 (rho = 0.547, *p* < 0.0001) and other DNA repair-related proteins such as RAD21 (rho = 0.529, *p* < 0.0001). Interestingly, ALDH1B1 exhibited a positive correlation with H2AX (rho = 0.288, *p* < 0.05), an important chromatin remodeling and DNA repair protein (Figure 7, Supplementary Materials, Table S3).

## 4. Discussion

ALDH1B1 has no clear physiological and pathophysiological roles, but recent studies have contributed to the better functional characterization of the protein. Specifically, ALDH1B1 has been found to be involved in β-cell development (in mice) [7], the maintenance of sperm motility (in horses) [24] and ethanol and retinaldehyde metabolism (in humans) [25]. Further studies have demonstrated that ALDH1B1 is associated with diabetes [26], colon cancer [2,8,9,13,14], pancreatic cancer [10,11] and osteosarcoma [27]. Want et al. showed that the expression of ALDH1B1 was significantly higher in colorectal adenomas and adenocarcinomas compared to normal and cancer-adjacent tissues [15]. Moreover, ALDH1B1 was found to be associated with cell morphology, proliferation, clonogenicity, enhanced chemoresistance, G2/M cell cycle arrest, EMT and migration in HT29 cells [2]. Matsumoto et al. exhibited increased ALDH1B1 and ALDH2 transcriptional and translational levels in human CRC cell lines and patient-derived tissues, indicating ALDH1B1’s critical role for colon cancer tumorigenesis [9]. Similarly, Singh et al., by utilizing shRNA-induced suppression of ALDH1B1 expression in a 3-D spheroid growth model and a nude mouse xenograft tumor model, demonstrated the crucial role of ALDH1B1 in formatting CRC tumors through the implication of CSC-related Wnt/β-catenin, Notch and PI3K/Akt signaling pathways [8].

The main aim of our study was to elucidate the role of ALDH1B1 in the DNA damage response process. For the purpose of the study, we utilized an isogenic HT29 cell line pair, differing only in the expression of ALDH1B1, as established previously [2]. The expression of ALDH1B1 in HT29 cells was correlated with the total and phosphorylated (at ser 15) p53 upregulation and enhanced resistance against the genotoxic and apoptotic effects of etoposide. Moreover, by utilizing the RT^2^ profiler™ PCR array, we demonstrated that HT29/ALDH1B1 cells exhibited differential expression of the genes implicated in DDR compared to HT29/mock control cells. Finally, the ALDH1B1 correlation with DDR-associated molecules, which are involved in base excision repair, nucleotide excision repair and NHEJ, was confirmed by analyzing colon and rectal adenocarcinoma patient samples obtained by TCGA.

It is known that DNA damage, and especially DSBs, results in the ATM-, ATR- and DNA-PK-dependent phosphorylation of histone H2AX at serine 139 residue (γH2Ax) [28,29,30] and in the subsequent formation of DNA foci in the nucleus [31]. Phosphorylation of H2AX is a key process in the DNA damage response, playing a crucial role in initiating the repair of DSBs [23]. The phosphorylated H2AX (ser139) is a critical factor for enhancing DNA accessibility, inducing, thus, the recruitment and accumulation of specific DNA damage response proteins at the DNA ends [23]. Furthermore, γH2AX triggers an epigenetic cascade, facilitates DSB rejoining and anchoring and induces the recruitment of numerous DSB repair proteins such as cohesins and MRN complex; it is considered a marker of DNA damage and repair. Our results demonstrated that the translational expression levels of γH2AX were up-regulated in ALDH1B1-overexpressing HT29 cells compared to the mock control under normal and etoposide conditions (1 h incubation). Interestingly, although DNA damage was found to be lower in the HT29/ALDH1B1 cells compared to HT29/mock cells, western blot results for the ser139 phosphorylated H2AX indicated enhanced H2AX phosphorylation in both normal and treated conditions (etoposide) in the ALDH1B1-overexpressing cells in comparison to mock control cells. Given that H2AX phosphorylation is considered as a marker of DNA damage, the increased levels of phosphorylated H2AX and increased survival observed in ALDH1B1-expressing cells would appear contradictory. A previous study revealed that constitutive phosphorylation of H2AΧ is related to the constitutive activation of ATM due to endogenous oxidative stress-induced DNA damage [32]. The activated-ATM monomers phosphorylate downstream targets such as p53 to regulate apoptosis, cell cycle arrest or DNA repair [23]. There are also inherent limitations in measuring H2AX phosphorylation as a measure of DNA and repair associated with the selection of appropriate time points and cell cycle phases [33,34]. The increased phosphorylation of H2AX observed in ALDH1B1-overexpressing cells may be a reflection of enhanced signaling leading to H2AX phosphorylation, which would result in a “state of cell alertness” by accelerating the recruitment of the DNA repair machinery, subsequently leading to less damage and better cell survival. Constitutive phosphorylation of H2AX at serine 139 residue under normal conditions is associated with cell cycle phase arrest [34]. Constitutive H2AX phosphorylation depends on the cell type and on the cell cycle phase, in which S-phase and G2/M-phase cells exhibit increased levels of γH2AX [35]. Accordingly, we demonstrated that the p53-dependent G2/M cell cycle arrest of HT29/ALDH1B1 cells [2] induces constitutive phosphorylation of H2AX, which may lead to an accelerated response to DNA damage and, subsequently, DNA repair. For this reason, we believe that the constitutive phosphorylation of H2AX in ALDH1B1-overexpressing cells is a significant finding that should be extensively studied in the future.

As ALDH1B1 appeared to be involved in DNA damage and repair response signaling in HT29 cells, we set out to analyze the expression pattern of DNA damage and repair response signaling genes and proteins on a wide scale by performing the RT^2^ profiler PCR array and assessing its correlation with DNA repair molecules using publicly available clinical samples. Our results demonstrated that ALDH1B1 altered the gene expression profile of DDS-related molecules in vitro and is statistically significantly positively correlated with DDR-related genes and/or proteins, which are involved in base excision repair (APEX1, FEN1, MPG, UNG and XRCC1), nucleotide excision repair (DDB1 and XPC) and NHEJ (CIB1, MRE11, PRKDC, RAD50, TP53BP1 and XRCC6).

Recent studies suggested that DDR-related pathways may be responsible for the chemo- and radio-resistance of CSCs [36]. An enhanced DDR may prevent CSCs and chemo-resistant cells from the chemotherapeutic or radiation pressure, enabling cancer metastasis [36]. CD133+ lung cancer cells exhibited the altered gene expression profile of chemotherapy-induced DNA repair molecules [37], whereas increased expression of DNA repair proteins and enhanced DDR was demonstrated in Lin-CD29^H^CD24^H^ mammary gland tumor-derived CSCs [38]. More specifically, the APEX1 signaling pathway plays a crucial role in regulating colon CSC growth, whereas FEN1, FANCG and RAD23B were up-regulated in chemo-resistant human colon cancer cell lines [39]. Furthermore, ALDH1A1-deficient (shALDH1A1) A2780/CP70 cells exhibited the downregulation of different DDR-related molecules such as FANCD2, FANCJ and XRCC1 [40]. Notably, ALDH1+ cells are correlated with radio-resistance in cervical carcinoma by activating the DNA damage checkpoint response and enhancing DNA repair ability [41].

The potential enhanced DDR of ALDH1B1-overexpressing colorectal adenocarcinoma cells may be one of the mechanisms that can relate with the cancer stem-like phenotype previously reported for ALDH1B1-overexpressing HT29 and CaCo2 cells. We suggest that ALDH1B1 could be implicated in DDR by altering the gene/protein expression profile of specific DDS-related molecules and/or facilitating the recruitment of DNA repair molecules. Quite interestingly, another member of the ALDH family, ALDH3A1, was reported to play an important role in the antioxidant defenses of cell homeostasis by maintaining DNA integrity through altering the expression profile of various DNA damage signaling-related genes in the human corneal epithelium [20]. ALDH1B1 may act upstream of the DNA-dependent protein kinase catalytic subunit (DNA-PKcs or PRKDC), which phosphorylates p53 at ser 15 and H2AX at ser139 residue (γH2AX), inducing a faster DNA damage response and repair process. This hypothesis needs to be further investigated and to be the object of study for future research.

## 5. Conclusions

In conclusion, HT29 ALDH1B1-overexpressing cells exhibited constitutive upregulation of the total and activated (phosphorylated at ser 15) p53, resistance against etoposide-induced DNA damage and apoptosis. ALDH1B1 is also associated with increased phosphorylation of H2AX (ser139) as well as with transcriptional upregulation of DNA repair molecules (MRE11A, PMS1, UNG) in HT29 cells. Bioinformatics analysis of 531 colon and rectal adenocarcinoma patient samples validated that ALDH1B1 is statistically significantly positively correlated with the upregulation of various DDS-related proteins. Our data raise an intriguing possibility that ALDH1B1 may in fact be able to sustain the CSC phenotype and promote growth of cancer cells by protecting cells from DNA damage. In this context, the selective suppression of ALDH1B1 and/or the use of specific ALDH1B1 inhibitors may be promising approaches for CSC-directed therapeutics in colon cancer. Further investigation is required to enhance our understanding on the underlined role of ALDH1B1 in DNA damage response and repair and its relation with cancer progression.

## Figures and Tables

**Table 1:** Median fluorescence intensity of total p53.

	HT29/Mock	HT29/ALDH1B1	Statistical Significance
Median Fluorescence intensity	579.5 ± 131.46	860.75 ± 118.71	*

Results are expressed as mean ± S.D. of three independent experiments. * *p* < 0.05.

**Table 2:** Percentage (%) of HT29/mock and HT29/ALDH1B1 cells in different stages of apoptosis under normal conditions and following etoposide treatment.

		Untreated	50 μΜ Etoposide	75 μΜ Etoposide
**HT29/mock** **(%)**	Live	83.03 ± 1.88	68.43 ± 3.12	59.39 ± 3.31
	Early apoptotic	8.32 ± 1.56	11.27 ± 2.53	12.19 ± 1.38
	Late apoptotic	7.78 ± 0.93	18.3 ± 1.93	22.82 ± 0.28
	Dead	0.87 ± 0.52	2.00 ± 1.15	5.60 ± 2.24
**HT29/ALDH1B1** **(%)**	Live	85.47 ± 0.65	75.97 ± 0.40	77.1 ± 1.47
	Early apoptotic	8.00 ± 0.54	12.03 ± 1.96	8.79 ± 0.97
	Late apoptotic	5.86 ± 0.30	11.00 ± 1.28	12.31 ± 0.56
	Dead	0.67 ± 0.62	1.00 ± 0.47	1.8 ± 0.12

Results are expressed as mean ± S.D. of three independent experiments.

## Data Availability

Part of the results presented in this study are based on data generated by the TCGA Research Network, which were downloaded from cBioportal.org (assessed on 1 February 2022).

## Supplementary Materials

The following supporting information can be downloaded at: https://www.mdpi.com/article/10.3390/cells11132017/s1, Table S1: List of genes included in the RT2 Profiler PCR Array for Human DNA Damage Signaling Pathway. Table S2: Correlation of mRNA levels between ALDH1B1 and DDS-related genes. Table S3: Correlation of protein levels between ALDH1B1 and DDS-related proteins.
