# Supplementary material for: Aldehyde Dehydrogenase 1B1 Is Implicated in DNA Damage Response in Human Colorectal Adenocarcinoma
Source: Cells. 2022 Jun 24;11(13):2017. doi: 10.3390/cells11132017 (PMC9265533; doi:10.3390/cells11132017)
Supplement: Supplementary file 1 [file cells-11-02017-s001.zip › cells-1754085-supplementary.pdf]

Supplementary Materials & Methods

Table S1. List of genes included in the RT² Profiler PCR Array for Human DNA Damage Signaling Pathway: Cat. no. 330231 PAHS-029ZA (Qiagen).

Position	UniGene	Genebank	Symbol	Description
A01	Hs.431048	NM_005157	ABL1	C-abl oncogene 1, non-receptor tyrosine kinase
A02	Hs.73722	NM_080649	APEX1	APEX nuclease (multifunctional DNA repair enzyme) 1
A03	Hs.367437	NM_000051	ATM	Ataxia telangiectasia mutated
A04	Hs.271791	NM_001184	ATR	Ataxia telangiectasia and Rad3 related
A05	Hs.694840	NM_032166	ATRIP	ATR interacting protein
A06	Hs.533526	NM_000489	ATRX	Alpha thalassemia/mental retardation syndrome X-linked
A07	Hs.591642	NM_000465	BARD1	BRCA1 associated RING domain 1
A08	Hs.624291	NM_004324	BAX	BCL2-associated X protein
A09	Hs.467020	NM_014417	BBC3	BCL2 binding component 3
A10	Hs.716515	NM_000057	BLM	Bloom syndrome, RecQ helicase-like
A11	Hs.194143	NM_007294	BRCA1	Breast cancer 1, early onset
A12	Hs.532799	NM_032043	BRIP1	BRCA1 interacting protein C-terminal helicase 1
B01	Hs.437705	NM_001789	CDC25A	Cell division cycle 25 homolog A (S. pombe)
B02	Hs.656	NM_001790	CDC25C	Cell division cycle 25 homolog C (S. pombe)
B03	Hs.184298	NM_001799	CDK7	Cyclin-dependent kinase 7
B04	Hs.370771	NM_000389	CDKN1A	Cyclin-dependent kinase inhibitor 1A (p21, Cip1)
B05	Hs.24529	NM_001274	CHEK1	CHK1 checkpoint homolog (S. pombe)
B06	Hs.291363	NM_007194	CHEK2	CHK2 checkpoint homolog (S. pombe)
B07	Hs.135471	NM_006384	CIB1	Calcium and integrin binding 1 (calmyrin)
B08	Hs.151573	NM_004075	CRY1	Cryptochrome 1 (photolyase-like)
B09	Hs.82201	NM_001896	CSNK2A2	Casein kinase 2, alpha prime polypeptide
B10	Hs.290758	NM_001923	DDB1	Damage-specific DNA binding protein 1, 127kDa
B11	Hs.700338	NM_000107	DDB2	Damage-specific DNA binding protein 2, 48kDa
B12	Hs.728989	NM_004083	DDIT3	DNA-damage-inducible transcript 3
C01	Hs.435981	NM_001983	ERCC1	Excision repair cross-complementing rodent repair deficiency, complementation group 1 (includes overlapping antisense sequence)
C02	Hs.487294	NM_000400	ERCC2	Excision repair cross-complementing rodent repair deficiency, complementation group 2
C03	Hs.498248	NM_130398	EXO1	Exonuclease 1
C04	Hs.567267	NM_000135	FANCA	Fanconi anemia, complementation group A
C05	Hs.208388	NM_033084	FANCD2	Fanconi anemia, complementation group D2
C06	Hs.591084	NM_004629	FANCG	Fanconi anemia, complementation group G
C07	Hs.409065	NM_004111	FEN1	Flap structure-specific endonuclease 1
C08	Hs.80409	NM_001924	GADD45A	Growth arrest and DNA-damage-inducible, alpha
C09	Hs.9701	NM_006705	GADD45G	Growth arrest and DNA-damage-inducible, gamma
C10	Hs.477879	NM_002105	H2AFX	H2A histone family, member X
C11	Hs.152983	NM_004507	HUS1	HUS1 checkpoint homolog (S. pombe)
C12	Hs.1770	NM_000234	LIG1	Ligase I, DNA, ATP-dependent
D01	Hs.432642	NM_002969	MAPK12	Mitogen-activated protein kinase 12
D02	Hs.35947	NM_003925	MBD4	Methyl-CpG binding domain protein 4
D03	Hs.709634	NM_024596	MCPH1	Microcephalin 1
D04	Hs.653495	NM_014641	MDC1	Mediator of DNA-damage checkpoint 1
D05	Hs.195364	NM_000249	MLH1	MutL homolog 1, colon cancer, nonpolyposis type 2 (E. coli)
D06	Hs.436650	NM_014381	MLH3	MutL homolog 3 (E. coli)
D07	Hs.459596	NM_002434	MPG	N-methylpurine-DNA glycosylase
D08	Hs.192649	NM_005590	MRE11A	MRE11 meiotic recombination 11 homolog A (S. cerevisiae)
D09	Hs.597656	NM_000251	MSH2	MutS homolog 2, colon cancer, nonpolyposis type 1 (E. coli)
D10	Hs.280987	NM_002439	MSH3	MutS homolog 3 (E. coli)
D11	Hs.492208	NM_002485	NBN	Nibrin
D12	Hs.66196	NM_002528	NTHL1	Nth endonuclease III-like 1 (E. coli)
E01	Hs.380271	NM_002542	OGG1	8-oxoguanine DNA glycosylase

E02	Hs.177766	NM_001618	PARP1	Poly (ADP-ribose) polymerase 1
E03	Hs.728886	NM_182649	PCNA	Proliferating cell nuclear antigen
E04	Hs.111749	NM_000534	PMS1	PMS1 postmeiotic segregation increased 1 (S. cerevisiae)
E05	Hs.632637	NM_000535	PMS2	PMS2 postmeiotic segregation increased 2 (S. cerevisiae)
E06	Hs.78016	NM_007254	PNKP	Polynucleotide kinase 3'-phosphatase
E07	Hs.591184	NM_003620	PPM1D	Protein phosphatase, Mg2+/Mn2+ dependent, 1D
E08	Hs.631593	NM_014330	PPP1R15A	Protein phosphatase 1, regulatory (inhibitor) subunit 15A
E09	Hs.491682	NM_006904	PRKDC	Protein kinase, DNA-activated, catalytic polypeptide
E10	Hs.531879	NM_002853	RAD1	RAD1 homolog (S. pombe)
E11	Hs.16184	NM_002873	RAD17	RAD17 homolog (S. pombe)
E12	Hs.375684	NM_020165	RAD18	RAD18 homolog (S. cerevisiae)
F01	Hs.81848	NM_006265	RAD21	RAD21 homolog (S. pombe)
F02	Hs.655835	NM_005732	RAD50	RAD50 homolog (S. cerevisiae)
F03	Hs.631709	NM_002875	RAD51	RAD51 homolog (S. cerevisiae)
F04	Hs.172587	NM_133509	RAD51B	RAD51 homolog B (S. cerevisiae)
F05	Hs.655354	NM_004584	RAD9A	RAD9 homolog A (S. pombe)
F06	Hs.546282	NM_002894	RBBP8	Retinoblastoma binding protein 8
F07	Hs.443077	NM_016316	REV1	REV1 homolog (S. cerevisiae)
F08	Hs.660132	NM_152617	RNF168	Ring finger protein 168
F09	Hs.485278	NM_183078	RNF8	Ring finger protein 8
F10	Hs.461925	NM_002945	RPA1	Replication protein A1, 70kDa
F11	Hs.369779	NM_012238	SIRT1	Sirtuin 1
F12	Hs.211602	NM_006306	SMC1A	Structural maintenance of chromosomes 1A
G01	Hs.81424	NM_003352	SUMO1	SMT3 suppressor of mif two 3 homolog 1 (S. cerevisiae)
G02	Hs.53454	NM_007027	TOPBP1	Topoisomerase (DNA) II binding protein 1
G03	Hs.654481	NM_000546	TP53	Tumor protein p53
G04	Hs.440968	NM_005657	TP53BP1	Tumor protein p53 binding protein 1
G05	Hs.697294	NM_005427	TP73	Tumor protein p73
G06	Hs.191334	NM_003362	UNG	Uracil-DNA glycosylase
G07	Hs.654364	NM_000380	XPA	Xeroderma pigmentosum, complementation group A
G08	Hs.475538	NM_004628	XPC	Xeroderma pigmentosum, complementation group C
G09	Hs.98493	NM_006297	XRCC1	X-ray repair complementing defective repair in Chinese hamster cells 1
G10	Hs.647093	NM_005431	XRCC2	X-ray repair complementing defective repair in Chinese hamster cells 2
G11	Hs.592325	NM_005432	XRCC3	X-ray repair complementing defective repair in Chinese hamster cells 3
G12	Hs.292493	NM_001469	XRCC6	X-ray repair complementing defective repair in Chinese hamster cells 6
H01	Hs.520640	NM_001101	ACTB	Actin, beta
H02	Hs.534255	NM_004048	B2M	Beta-2-microglobulin
H03	Hs.592355	NM_002046	GAPDH	Glyceraldehyde-3-phosphate dehydrogenase
H04	Hs.412707	NM_000194	HPRT1	Hypoxanthine phosphoribosyltransferase 1
H05	Hs.546285	NM_001002	RPLP0	Ribosomal protein, large, P0
H06	N/A	SA_00105	HGDC	Human Genomic DNA Contamination
H07	N/A	SA_00104	RTC	Reverse Transcription Control
H08	N/A	SA_00104	RTC	Reverse Transcription Control
H09	N/A	SA_00104	RTC	Reverse Transcription Control
H10	N/A	SA_00103	RTC	Positive PCR Control
H11	N/A	SA_00103	RTC	Positive PCR Control
H12	N/A	SA_00103	RTC	Positive PCR Control

Table S2. Correlation of mRNA levels between ALDH1B1 and DDS-related genes.

Gene Name	Rho	P-value	Statistical significance
ABL1	0.284	0.00000000414	****
APEX1	0.124	0.004224	**
ATM	-0.051	0.243874	-
ATR	0.045	0.296027	-
ATRIP	0.073	0.090802	-
ATRX	0.011	0.802513	-

BARD1	-0.075	0.085043	-
BAX	0.074	0.089518	-
BBC3	0.049	0.255766	-
BLM	-0.053	0.226768	-
BRCA1	-0.035	0.415595	-
BRIP1	-0.043	0.324472	-
CDC25A	0.094	0.029881	*
CDC25C	-0.022	0.610128	-
CDK7	0.021	0.626746	-
CDKN1A	-0.121	0.00531	**
CHEK1	0.003	0.935879	-
CHEK2	0.033	0.447983	-
CIB1	-0.041	0.343212	-
CRY1	-0.218	0.000000387	****
CSNK2A2	0.110	0.010848	*
DDB1	-0.031	0.472816	-
DDB2	0.016	0.718415	-
DDIT3	-0.009	0.838622	-
ERCC1	0.031	0.471	-
ERCC2	0.053	0.223188	-
EXO1	-0.123	0.004595	**
FANCA	-0.086	0.047241	*
FANCD2	-0.103	0.017635	*
FANCG	0.085	0.049217	*
FEN1	0.074	0.086874	-
GADD45A	-0.062	0.150554	-
GADD45G	0.170	0.000084	****
H2AX	-0.009	0.843025	-
HUS1	0.050	0.252429	-
LIG1	0.007	0.869712	-
MAPK12	-0.208	0.00000128	****
MBD4	0.007	0.880751	-
MCPH1	-0.042	0.339783	-
MDC1	-0.069	0.111929	-
MLH1	0.153	0.000407	***
MLH3	0.035	0.415834	-
MPG	0.036	0.411011	-
MRE11	0.041	0.340112	-
MSH2	-0.041	0.349813	-
MSH3	0.011	0.793598	-
NBN	-0.119	0.00584	**
NTHL1	0.237	0.0000000325	****
OGG1	0.155	0.000341	***
PARP1	-0.081	0.063085	-
PCNA	0.109	0.012343	*
PMS1	-0.012	0.791036	-
PMS2	-0.008	0.850413	-
PNKP	0.021	0.633336	-
PPM1D	-0.018	0.681547	-
PPP1R15A	-0.118	0.006495	**
PRKDC	-0.036	0.411331	-
RAD1	0.018	0.674291	-
RAD17	-0.073	0.094466	-
RAD18	-0.076	0.078201	-
RAD21	-0.179	0.0000329	****
RAD50	0.090	0.03874	*
RAD51	-0.018	0.683691	-
RAD51B	0.031	0.48219	-
RAD9A	-0.114	0.008724	**
RBBP8	-0.208	0.00000132	****
REV1	0.015	0.736747	-
RNF168	-0.067	0.124754	-
RNF8	0.033	0.449283	-
RPA1	-0.052	0.234589	-
SIRT1	-0.090	0.037457	*

SMC1A	0.062	0.152291	-
SUMO1	0.038	0.377362	-
TOPBP1	-0.091	0.035844	*
TP53	0.143	0.000965	***
TP53BP1	0.129	0.002863	**
TP73	-0.204	0.00000213	****
UNG	0.121	0.005064	**
XPA	0.152	0.000441	***
XPC	0.062	0.155427	-
XRCC1	0.113	0.009416	**
XRCC2	-0.043	0.32225	-
XRCC3	-0.044	0.306193	-
XRCC6	0.003	0.939439	-

*$p < 0.05$, **$p < 0.01$, ***$p < 0.001$, ****$p<0.0001$.

Table S3. Correlation of protein levels between ALDH1B1 and DDS-related proteins.

Protein Name	Rho	P-value	Statistical significance
APEX1	0.615	0.00000000274	****
ATM	0.007	0.953844	-
ATRX	0.187	0.104299	-
BAX	0.464	0.0000214	****
CDK7	0.002	0.989514	-
CHEK2	-0.004	0.972997	-
CIB1	0.330	0.003407	**
CSNK2A2	0.324	0.003993	**
DDB1	0.556	0.000000149	****
DDB2	0.066	0.570772	-
ERCC2	-0.074	0.519907	-
FANCD2	-0.090	0.436974	-
FEN1	0.420	0.000143	***
H2AX	0.288	0.011211	*
LIG1	0.158	0.169384	-
MDC1	0.077	0.506527	-
MLH1	0.116	0.313628	-
MPG	0.445	0.0000506	****
MRE11	0.604	0.00000000627	****
MSH2	0.234	0.040617	*
MSH3	-0.223	0.051539	-
NBN	0.314	0.005351	**
PARP1	0.286	0.011822	*
PCNA	0.337	0.002693	**
PNKP	0.310	0.00602	**
PRKDC	0.584	0.0000000247	****
RAD21	0.529	0.000000765	****
RAD50	0.532	0.000000641	****
RPA1	0.418	0.000154	***
SMC1A	0.410	0.000209	***
SUMO1	0.066	0.571117	-
TP53	-0.022	0.846748	-
TP53BP1	0.351	0.001744	**
UNG	0.275	0.015645	*
XPC	0.296	0.009038	**
XRCC1	0.395	0.00038	***
XRCC6	0.547	0.000000265	****

*$p < 0.05$, **$p < 0.01$, ***$p < 0.001$, ****$p<0.0001$.
